# Dexamethasone suppression for ^18^F-FDG PET/CT to localize ACTH-secreting pituitary tumors

**DOI:** 10.1186/s40644-023-00600-8

**Published:** 2023-09-12

**Authors:** Kyungwon Kim, Dong Kyu Kim, Ju Hyung Moon, Eui Hyun Kim, Sun Ho Kim, Cheol Ryong Ku, Eun Jig Lee

**Affiliations:** 1 Endocrinology, Institute of Endocrine Research, Department of Internal Medicine, Yonsei University College of Medicine, Seoul, Republic of Korea; 2 Department of Radiology, Severance Hospital, Yonsei University College of Medicine, Seoul, Republic of Korea; 3 Department of Neurosurgery, Severance Hospital, Yonsei University College of Medicine, Seoul, Republic of Korea

**Keywords:** ^18^F-FDG PET/CT, ACTH-secreting pituitary tumor, Cushing’s disease, Dexamethasone suppression, High-dose dexamethasone suppression test

## Abstract

**Background:**

^18^Fluorine-Fluoro-deoxy-glucose (^18^F-FDG) positron emission tomography (PET) is widely used for diagnosing various malignant tumors and evaluating metabolic activities. Although the usefulness of ^18^F-FDG PET has been reported in several endocrine diseases, studies on pituitary disease are extremely limited. To evaluate whether dexamethasone (DEX) suppression can improve ^18^F-FDG PET for the localization of adrenocorticotropic hormone-secreting adenomas in the pituitary gland in Cushing’s disease (CD).

**Methods:**

We included 22 patients with CD who underwent PET imaging before and after DEX administration. We compared the success rates of PET before and after DEX suppression, magnetic resonance imaging (MRI), and bilateral inferior petrosal sinus sampling (BIPSS). We determined the final locations of adenomas based on intraoperative multiple-staged resection and tumor tissue identification using frozen sections. Standardized uptake value (SUV) were analyzed to confirm the change of intensity of adenomas on PET.

**Results:**

Twenty-two patients were included (age at diagnosis: 37 [13–56] years), and most were women (90.91%). Pituitary adenomas compared to normal pituitaries showed increased maximum SUV after DEX suppression but without statistical significance (1.13 versus. 1.21, z=-0.765, *P* = 0.444). After DEX suppression, the mean and maximum SUV of adenomas showed a positive correlation with nadir cortisol levels in high-dose DEX suppression test (Rho = 0.554, *P* = 0.007 and Rho = 0.503, *P* = 0.017, respectively). In reference sites, mean SUV of cerebellum was significantly decreased (7.65 vs. 6.40, *P* = 0.006^*^), but those of the thalamus and gray matter was increased after DEX suppression (thalamus, 8.70 vs. 11.20, *P* = 0.010^*^; gray matter, 6.25 vs. 7.95, *P* = 0.010^*^).

**Conclusion:**

DEX suppression did not improve ^18^F-FDG PET/CT localization in patients with CD.

**Supplementary Information:**

The online version contains supplementary material available at 10.1186/s40644-023-00600-8.

## Introduction

Cushing’s disease (CD) is a rare endocrine disease that results from chronic exposure to high cortisol levels because of adrenocorticotropic hormone (ACTH)-secreting pituitary tumors and is associated with increased morbidity and mortality. It represents approximately 80% of all cases of endogenous hypercortisolism [1–3]. Accurate localization of primary lesions in CD leads to improved remission rates and reduced adverse events following surgery [4, 5]. A biochemical remission rate of 90–100% has been reported when tumors are localized before surgery, but it can decrease to 50–60% when surgery is performed when the location of the tumor is unknown in patients with CD [6–8].

Currently, magnetic resonance imaging (MRI) is the gold standard for detecting pituitary adenomas. Nevertheless, modern MRI modalities, including dynamic or volumetric sequences, can reliably detect corticotrophic adenomas in 50–90% cases of CD [9–12]. This indicates that complementary imaging strategies are required to improve the localization of primary lesions in CD.

One of the most characteristic features of corticotrophic adenomas is a compromised response to negative glucocorticoid feedback, which defines glucocorticoid resistance [13]. ACTH activates the adrenal glands to synthesize and secrete cortisol, which in turn negatively modulates the release of ACTH from the pituitary gland and corticotrophin-releasing hormone (CRH) and vasopressin from the hypothalamus [1]. In CD, a corticotrophic tumor is only partially sensitive to the inhibitory feedback exerted by cortisol, which in turn is not regulating its own production and secretion of ACTH, resulting in both excessive ACTH and cortisol levels. Glucocorticoid resistance is caused by multiple factors including glucocorticoid receptor availability, splice variant expression and affinity, and imbalanced glucocorticoid receptor signaling [14, 15].

Radioactive ^18^ F-fluorodeoxyglucose positron emission tomography/computed tomography (^18^F-FDG PET/CT) often demonstrates increased fluorodeoxyglucose (FDG) uptake in nonfunctioning and hormone-secreting pituitary adenomas [16–18]. In large observational studies of whole-body ^18^F-FDG positron emission tomography (PET) scans, incidental sellar ^18^F-FDG uptake was found in < 1% of cases, and this sign is highly specific for pituitary adenomas [19–21]. ^18^F-FDG PET imaging can detect up to 40% of corticotropinomas, some as small as 3 mm, and the rate of PET detection of corticotropinomas can be increased by CRH stimulation [9, 22].

Here, we evaluated whether DEX suppression could improve the localization of ACTH-secreting adenomas using ^18^F-FDG PET/CT in patients with CD. The rationale for this is as follows. FDG uptake of corticotrophic adenomas is less suppressed than that of normal pituitary glands after DEX suppression due to glucocorticosteroid resistance.

## Materials and methods

### Study design and population

In this retrospective cohort study, we enrolled all patients with CD who underwent two rounds of ^18^F-FDG-PET/CT before and after 8-mg DEX suppression and pituitary MRI before surgery. Total 22 patients were included in this study, of which thirteen had bilateral inferior petrosal sinus sampling (BIPSS) results. All patients were diagnosed with CD by staff of the Department of Endocrinology and/or Neurosurgery at Severance Hospital between 2014 and 2015. The diagnosis of CD was confirmed based on biochemical test results, including the cortisol, 24-hour urine free cortisol (24 h UFC), and serum ACTH levels, overnight dexamethasone suppression test (ON DST) results, and high-dose dexamethasone suppression test (HD DST) results.

Immediate remission was defined as hypocortisolism (serum cortisol level < 1.8 µg/dL) within the first 7 days after surgery. Delayed remission was defined as the achievement of hypocortisolism within 6 months, although immediate remission was not confirmed. If patients showed elevated postoperative cortisol levels and needed additional treatment within 6 months after surgery, we defined them as having persistent disease [23–25].

A serum cortisol concentration > 1.8 µg/dL for 8 h in the morning after 1 mg of DEX was given at midnight was considered to be a positive result in the ON DST [26]. Suppression of the serum cortisol level by > 50% for 6 h after 2 mg of DEX was administered for 2 days was defined as the suppression on the HD DST [26]. The final diagnosis was confirmed using surgical pathology and clinical follow-up.

### Endocrinological evaluation

All laboratory analyses were performed at the Department of Laboratory Medicine, Severance Hospital. Preoperative cortisol and 24 h UFC were measured by chemiluminescence immunoassay using an automated UniCel DXC880i Synchron analyzer (Beckman Coulter, Pasadena, CA, USA; coefficient of variation [CV] ± 15 nmol/L at < 100 nmol/L and ± 15% at > 100 nmol/L). Preoperative ACTH levels were analyzed by electrochemiluminescence immunoassay using the Roche Cobas 6000 analyzer (Roche Diagnostics GmbH, Mannheim, Germany; CV ± 2.0 pmol/L at < 20 pmol/L and ± 10% at > 20 pmol/L).

The serum cortisol concentration at 8:00 am the following day after 1 mg of DEX was administered at midnight was considered positive on the ON DST. We determined the result as “suppression” by the cortisol level of < 1.8 µg/dL. A serum cortisol level suppressed by > 50% of the original level after 6 hourly administrations of 2 mg of DEX for 48 h was defined as suppression on the HD DST [27].

### ^18^F-FDG PET/CT evaluation

PET/CT was performed using a GEADVANCE PET scanner (GE, Milwaukee, WI, USA) after the intravenous injection of 7–9 mCi of ^18^F-FDG. All patients fasted for at least 6 h before the test. Emission scanning was continued for 15 min (4.25-mm axial spatial resolution, 4.8-mm transaxial spatial resolution). Transmission scans were performed for 8 min using triple Ge-68 rod sources to correct attenuation. Gathered data were reconstructed in a 128 × 128 × 35 matrix with a pixel size of 1.95 × 1.95 × 4.25 mm by means of a filtered back-projection algorithm employing a transaxial 8.5-mm Hanning filter and 8.5-mm axial ramp filter. Two specialists independently interpreted the encoded baseline PET images, and after a two-week period, they interpreted the encoded post DEX suppression PET images. Each specialist was blinded to MRI imaging, clinical characteristics, and surgical outcomes of these subjects. Each was tasked with determining whether the PET image indicated a “negative” or “positive” result for pituitary adenoma and its location on a high-resolution computer screen.

The scan after DEX suppression was performed 24 h after the oral administration of 8 mg of DEX using the same procedures as for the baseline PET/CT scan.

### ^18^F-FDG uptake analysis

**Fig. 1:**
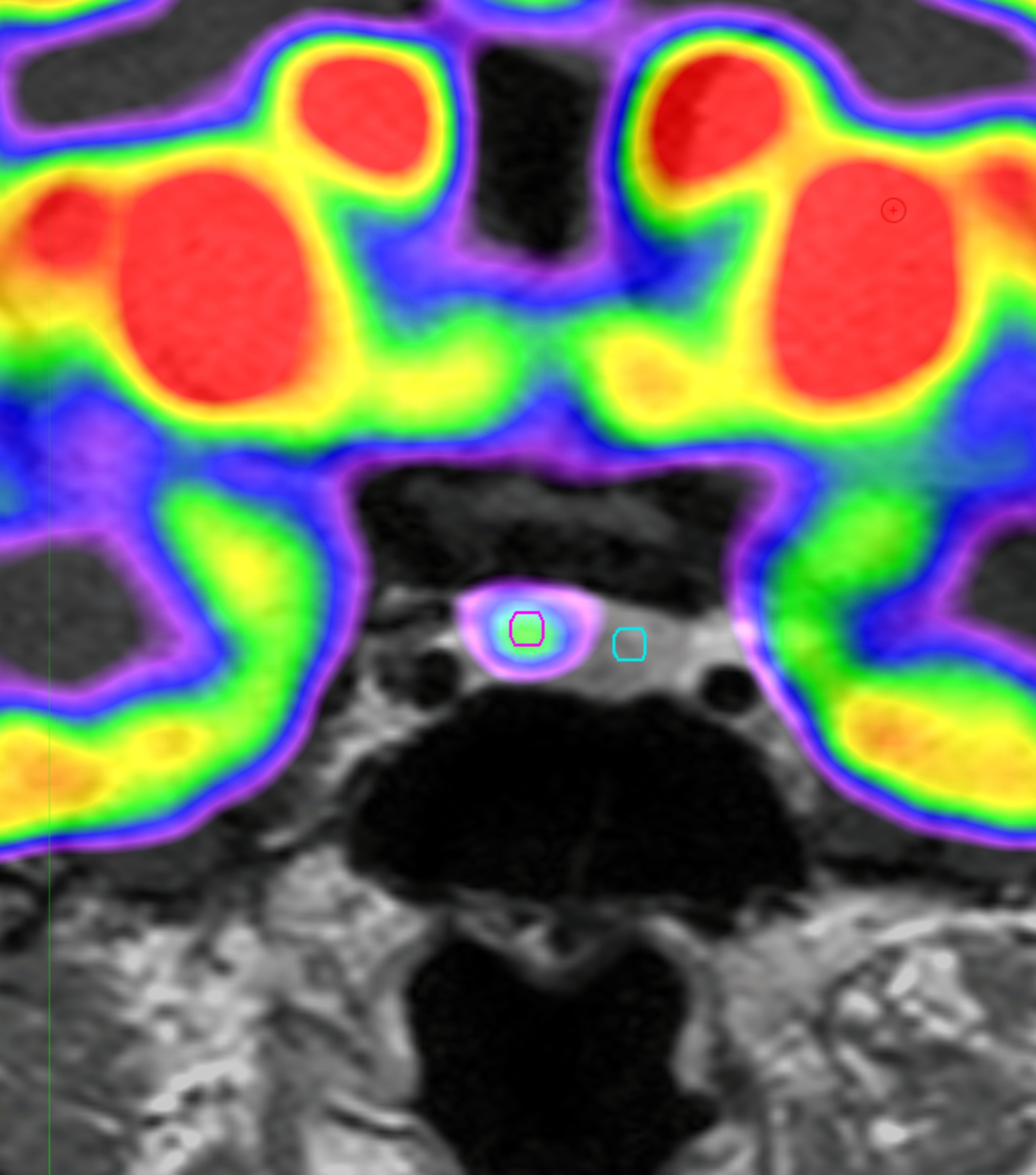
Images of ROI for pituitary adenoma on^18^F-FDG PET scan Example of ROI definition in pituitary adenomas of ^18^F-FDG PET scan of the patients with CD. We draw the fixed circular ROI with a 3-mm diameter for pituitary adenomas (red circle) and normal pituitary gland (green circle) ROI, Reason of interest; ^18^F-FDG PET, ^18^ F-fluorodeoxyglucose positron emission tomography; CD, Cushing disease

The Region of interest (ROI) was drawn using MIM software (version 6.5, Software INc., Cleveland, OH, USA) (Fig. 1). PET images were reviewed by experienced by an experienced specialist. The pituitary gland was identified and a circular ROI was drawn. A fixed ROI with a 3-mm diameter was used for all patients. The ROI was placed on the lesion with the highest FDG uptake. If there was no significantly increased FDG uptake, the same sized circular ROI was drawn on the suspected adenoma location. For the normal pituitary gland, the same sized 3 mm ROI was used.

The mean standardized uptake value (SUV_mean_) and maximum SUV (SUV_max_) for pituitary adenomas and normal pituitary glands were automatically measured using MIM, version 6.5 (Software Inc., Cleveland, OH, USA). The standardized uptake value (SUV) of the volume of interest was calculated as follows: (decoy-corrected activity (kBq) / volume (mL)) / (injected dose (kBq) / body weight (g)).

SUV_mean_ and SUV_max_ of pituitary adenomas were divided into the SUV_mean_ of normal pituitary glands for adjustment. We used the ratio of SUV_max_ to SUV_mean_ to analyze the homogeneity of the pituitary adenomas.

### MRI evaluation

All patients underwent pituitary MRI with a 3.0-Tesla scanner (Achieva, Philips Medical Systems, Best, the Netherlands). Imaging protocols included T1-weighted imaging, T2-weighted imaging, and delayed gadolinium-enhanced T1-weighted imaging. The extent, location, and sizes of the pituitary tumors were reviewed based on official records determined by radiologists.

Pituitary tumors were classified based on radiological findings using MRI of the sellar and parasellar regions. Type I refers to tumors < 1 cm in diameter limited to the sella. Type II tumors extend into the suprasellar space, < 1 cm from the diaphragm. Type III includes tumors extending into the suprasellar space > 1 cm from the diaphragm or sphenoid sinus and encroaching on the internal carotid arteries. Lastly, type IV refers to adenomas with obvious invasion into the cavernous sinus, as shown on MRI, and into the medial dural wall of the cavernous sinus, as confirmed during surgery.

### BIPSS

Before surgery, BIPSS was performed to confirm the cause of CD and lateralize the tumors. A catheter was placed in patients using a unilateral femoral venous approach and 3 cc of blood was collected from the peripheral (P) and both inferior petrosal sinuses (IPS) [28]. CRH at a dose of 1 µg/kg was administered, and peripheral and petrosal samples were drawn after 5 and 10 min, respectively. The catheters and sheath were removed, and the groin was compressed under pressure until venous hemostasis was achieved.

The IPS:P prolactin ratio was calculated at each time point to confirm the accuracy of the inferior petrosal venous sampling. A value of ≥1.8 was considered successful IPS catheterization. The prolactin-normalized ACTH ratio was calculated by dividing the dominant ACTH IPS:P ratio by the concurrent and ipsilateral IPS:P prolactin ratio. A value of ≥1.3 was considered diagnostic of CD. An intersinus ACTH ratio of ≥1.4 either at baseline or after stimulation was used for lateralization of the pituitary adenoma [29].

### Location of the adenoma

The final assignment of the true location of the pituitary adenoma was based on intraoperative multiple stage resection and tumor tissue identification using frozen sections. Surgically identified adenomas were histologically evaluated and stained for ACTH immunoreactivity. In cases of multiple specimens obtained during the procedure, the true location of the adenoma was assigned based on the original site of the specimen containing the adenoma [30].

### Statistical analysis

Data are presented as medians (ranges) or numbers (percentages). The baseline characteristics of the patients were compared using Kruskal–Wallis’ test with Dunn’s procedure for nonparametric continuous variables. Categorical variables were compared using Fisher’s exact test. Spearman’s correlation coefficients were used to determine the correlation between FDG uptake and hormone levels. Wilcoxon’s signed-rank test was used to identify changes in the SUV after DEX administration.

The interobserver agreement for image analysis was assessed using κ statistics. κ values were categorized as follows: κ < 0.20 indicated poor agreement, κ of 0.21–0.40 indicated fair agreement, κ of 0.41–0.60 indicated moderate agreement, κ of 0.61–0.80 indicated good agreement, and κ > 0.81 indicated excellent agreement [31].

Statistical significance was set at a two-sided *P* < 0.05. All statistical analyses were performed using SPSS software (IBM Corp., Armonk, NY, USA).

## Results

### Patient characteristics

We enrolled all patients with CD who underwent two rounds of the ^18^F-FDG PET/CT with or without DEX suppression and sellar MRI before transsphenoidal adenectomy (TSA). Twenty-two patients were included (age at diagnosis: 37 [13–56] years), and most were women (90.91%). Patients’ baseline characteristics are shown in Table 1. There were 16 microadenomas and 6 macroadenomas. Immediate remission was achieved in 81.82% of the patients and delayed remission in 13.64%; one patient showed persistent disease after TSA. The median preoperative 24 h UFC, serum ACTH, and cortisol levels were 443.35 (93.00–4452.00) µg/day, 36.16 (6.00–92.00) pg/mL, and 18.55 (6.00–40.00) µg/dL. The size of pituitary adenomas on MRI was 7.85 (2.00–28.00) mm. The Ki-67 index of 47.06% of adenomas ranged from 1 to 2, that of 35.29% was below 1, and that of 17.65% was 2 or higher. Overall, 75.00% of the adenomas were classified as Knosp grade 0, 5.00% as grade 1, 5.00% as grade 3b, and 15.00% as grade 4. In total, 77.27% (17/22) of patients had an ACTH-staining adenoma. Only one patient showed unsuppressed cortisol levels on the HD DST.

**Table 1:** Patients’ imaging and clinical characteristics

	Total (N = 22)
Age at diagnosis (years)	37.50 (13.00–56.00)
Sex	
Male (%)	2/22 (9.09%)
Female (%)	20/22 (90.91%)
24 h UFC (µg/day)	443.35 (93.20–4452.20)
Preop ACTH level (pg/mL)	36.16 (5.69–91.82)
Preop cortisol level (µg/dL)	18.55 (5.50–40.40)
Nadir cortisol on the HD DST (µg/dL)	2.40 (0.40–14.36)
Suppression on the 1-mg ON DST	0/22 (0.00%)
Suppression on the HD DST	21/22 (95.45%)
Result of TSA surgery	
Remission (%)	18/22 (81.82%)
Delayed remission (%)	3/22 (13.64%)
No remission (%)	1/22 (4.56%)
Size on MRI (mm)	7.85 (2.00–28.00)
Ki-67	
< 1	6/17(35.29%)
1–2	8/17(47.06%)
≥ 2	3/17(17.65%)
Knosp classification (%)	
0	15/20 (75.00%)
1	1/20 (5.00%)
2	0/20 (0.00%)
3a/b	1/20 (5.00%)
4	3/20 (15.00%)
**Baseline** ^**18**^ **F-FDG PET/CT**	
Adenoma SUV_mean_	4.60(2.80–8.30)
Adenoma SUV_max_	5.05(3.20–8.60)
^**18**^ **F-FDG PET/CT after DEX suppression**	
Adenoma SUV_mean_	4.50(2.10–7.00)
Adenoma SUV_max_	4.70(2.50–7.50)

Success group, patients with CD whose tumors were successfully localized by ^18^F-FDG PET/CT after DEX suppression; Failed group, patients with CD whose tumors were not localized by ^18^F-FDG PET/CT after DEX suppression; 24 h UFC, 24-hour urine free cortisol; ACTH, adrenocorticotropic hormone; MRI, magnetic resonance imaging; ON DST, overnight dexamethasone suppression test; HD DST, high-dose dexamethasone suppression test; CD, Cushing’s disease; SUV_mean_, mean standardized uptake value; SUV_max_, maximum standardized uptake value; ^18^F-FDG-PET/CT, ^18^ F-fluorodeoxyglucose positron emission tomography/computed tomography; Preop, preoperative

### MRI negative but PET positive case

**Fig. 2:**
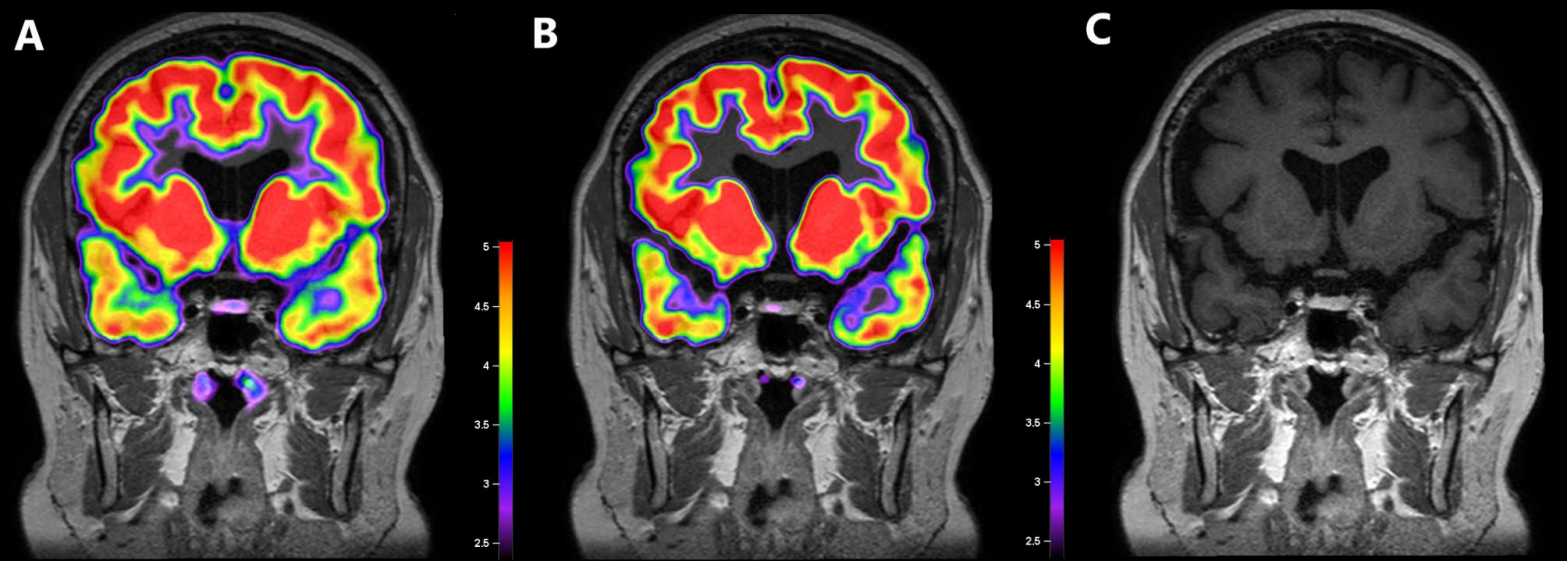
Images of a corticotroph with negative MRI but positive^18^ F-FDG PET/CT after DEX suppression An MRI-negative adenoma was detected on ^18^F-FDG PET/CT at baseline and after DEX suppression. In this patient, the pituitary adenoma was visible on PET scans at baseline (**B**) and after DEX suppression (**C**) at the same location, as confirmed by the surgeon **A**. Co-registered baseline ^18^F-FDG PET/CT and MRI images. Diffuse ^18^F-FDG uptake is detected in the pituitary fossa with an SUV_mean_ of 0.86 and SUV_max_ of 1.03, but there was failure to localize the adenoma on baseline ^18^F-FDG PET/CT. **B**. Co-registered ^18^F-FDG PET/CT and MRI images after DEX suppression. ^18^F-FDG uptake is not suppressed in the right side of the pituitary gland with an SUV_mean_ of 1.03 and SUV_max_ of 1.06. ^18^F-FDG PET/CT after DEX suppression was successful in localizing the right-sided corticotrophic adenoma **C**. MRI image. There is no suspicious lesion in the pituitary gland ACTH, adrenocorticotropic hormone; MRI, magnetic resonance imaging; ^18^F-FDG, ^18^ F-fluorodeoxyglucose; PET/CT, positron emission tomography/computed tomography; DEX, dexamethasone; SUV_mean_, mean standardized uptake value; SUV_max_, maximum standardized uptake value

Two patients showed negative MRI results, and one of them showed FDG uptakes on both ^18^F-FDG PET scans at baseline and after DEX suppression. A 26-year-old man visited our hospital complaining of weight gain and was diagnosed with ACTH-dependent CD. Cortisol secretion was suppressed on the HD DST; however, sellar MRI did not reveal any suspicious lesions. BIPSS revealed a central tumor (central/peripheral ACTH level of 36.25 after CRH stimulation) lateralized to the right side of the pituitary gland. The patient underwent ^18^F-FDG-PET/CT before and after DEX suppression to identify the primary lesions. Baseline PET/CT showed diffused FDG uptake with an SUV_max_ of 1.03 at the pituitary fossa but failed to localize the tumor. After DEX treatment, focal FDG uptake with an SUV_max_ of 1.06 remained at the right side of the pituitary fossa, which resulted in the successful localization of the corticotrophic adenoma. The MRI and PET/CT images of this case are presented in Fig. 2A–C. During TSA, the surgeon identified solid tumor-like tissues on the right side of the pituitary gland and successfully removed them. Results of pathology and ACTH immunohistochemistry were negative, but the patient achieved immediate biochemical remission and CD-related symptoms were relieved after surgery. We followed the patient for 98 months after the surgery and confirmed that he had lived without recurrence.

### Change of ^18^F-FDG uptake after DEX suppression

**Fig. 3:**
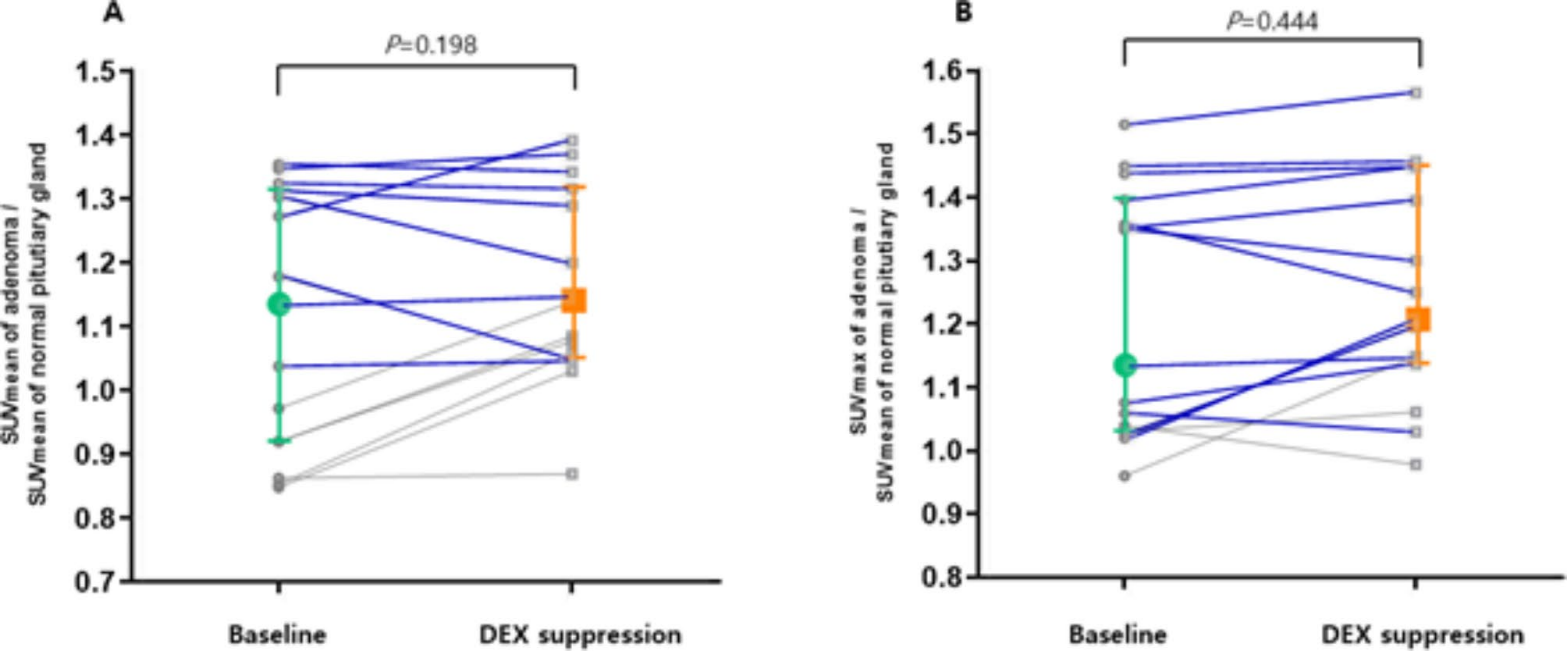
Changes in the SUVs of corticotrophs between^18^F-FDG PET/CT before and after DEX suppression The SUV_mean_ (**A**) and SUV_max_ (**B**) of corticotrophic adenomas are shown in this pairwise analysis. The SUV_mean_ did not changed after DEX suppression from (z=-1.288, *P* = 0.198). The SUV_max_ of the corticotrophic adenoma increased from 1.13 to 1.21 (z=-0.765, *P* = 0.444). In this analysis, the SUV_mean_ and SUV_max_ of pituitary adenomas were adjusted using the SUV_mean_ of the normal pituitary gland. Colored plots and bars presented median and interquartile range in this figure. We presented the tumors with size larger than 5 mm and SUV adjusted by normal pituitary>1 for blue line SUV_mean_, mean standardized uptake value; SUV_max_, maximum standardized uptake value; DEX, dexamethasone; ^18^F-FDG, ^18^ F-fluorodeoxyglucose; PET/CT, positron emission tomography/computed tomography

We included 18 pituitary adenomas that were successfully localized using PET/CT after DEX suppression, and analyzed the change of SUV for 15 adenomas, excluding outliers with SUV over 2.00. The results are presented in Fig. 3A and B. The SUV_mean_ of adenomas did not changed after DEX suppression compared to normal pituitary glands (SUV_mean_ of adenoma/SUV_mean_ of normal pituitary glands: 1.13 [0.85–1.35] vs. 1.14 [0.87–1.39], z=-1.288, *P* = 0.198). DEX suppression increased SUV_max_ of adenomas compared to normal pituitary glands but without statistical significance (SUV_max_ of adenoma/SUV_mean_ of normal pituitary glands: 1.13 [0.96–1.52] vs. 1.21 [0.97–1.56], z=-0.765, *P* = 0.444).

In Fig. 3, the blue line indicates change in SUV of adenomas larger than 5 mm with higher FDG uptake than the surrounding pituitary parenchyma. For these adenomas, DEX suppression did not change the SUV (SUV_mean_ of adenoma/SUV_mean_ of normal pituitary glands: 1.31 [1.04–2.52] vs. 1.33 [1.05–2.38], z=-0.784, *P* = 0.433; SUV_max_ of adenoma/SUV_mean_ of normal pituitary glands: 1.36 [1.02–2.61] vs. 1.40 [1.03–2.65], z=-1.022, *P* = 0.307).

The value of SUV increased in 73.33% adenomas, while the SUV_max_ increased in 66.67% compared with normal pituitary glands after DEX treatment.

### Correlation between the hormone level and ^18^F-FDG uptake

Table 2 shows the results of the Spearman correlation analysis of the SUV with preoperative cortisol, ACTH, and nadir cortisol levels on the HD DST. On the baseline ^18^F-FDG PET scan, the SUV_max_ of the adenomas did not show any correlation with the levels of three hormones. The SUV_mean_ of adenomas showed a positive correlation with nadir cortisol levels on the HD DST (*P* = 0.014) and preoperative ACTH levels, with marginal significance (*P* = 0.062). After DEX suppression, the SUV_max_ and SUV_mean_ of adenomas had a positive correlation with moderate degrees of nadir cortisol on the HD DST (SUV_max_: Spearman Rho = 503, *P* = 0.017; SUV_mean_: Spearman Rho = 0.554, *P* = 0.007).

**Table 2:** Correlation between FDG uptakes and hormone levels

	Spearman correlation coefficient (Rho), *P*-value		
	Adenoma SUV_max_/normal pituitary SUV_mean_		
	Baseline		DEX suppression
Preoperative cortisol	Rho = 1.121, *P* = 0.633	Rho = 0.299, *P* = 0.227	
Preoperative ACTH	Rho = 0.267, *P* = 0.284	Rho = 0.218, *P* = 0.385	
Nadir cortisol on the HD DST	Rho = 0.304, *P* = 0.168	Rho = 0.503, *P* = 0.017	
	**Adenoma SUV**_**mean**_ **/normal pituitary SUV**_**mean**_		
	**Baseline**		**DEX suppression**
Preoperative cortisol	Rho = 0.262, *P* = 0.293	Rho = 0.389, *P* = 0.111	
Preoperative ACTH	Rho = 0.448, *P* = 0.062	Rho = 0.313, *P* = 0.206	
Nadir cortisol on the HD DST	Rho = 0.516, *P* = 0.014^*^	Rho = 0.554, *P* = 0.007^*^	

SUV_mean_, mean standardized uptake value; SUV_max_, maximum standardized uptake value; DEX, dexamethasone; ACTH, adrenocorticotropic hormone; DST, high-dose dexamethasone suppression test; FDG, fluorodeoxyglucose

### FDG uptake of reference sites after DEX suppression

We evaluated the FDG uptake for five types of reference areas (normal pituitary gland, cerebellum, thalamus, white matter, and gray matter) (Table 3). Normal pituitary gland and white matter did not affect the unadjusted SUV_mean_ by DEX suppression (all *P* >0.05). DEX significantly increased SUV_mean_ of the thalamus and gray matter (thalamus, 8.70 [4.40–22.70] vs. 11.20 [6.40–17.5], *P* = 0.010^*^; gray matter, 6.25 [2.50–15.00] vs. 7.95 [5.00–11.90], *P* = 0.010^*^). However, SUV_mean_ of the cerebellum significantly decreased after DEX administration (7.65 [4.50–10.80] vs. 6.40 [2.60–12.00], *P* = 0.006^*^).

**Table 3:** The change of FDG uptake for reference sites after DEX suppression in the patients with CD

SUV_mean_	Baseline ^18^F-FDG PET/CT	DEX suppression ^18^F-FDG PET/CT	Wilcoxon Z	*P* value
Normal pituitary gland	3.50(2.40–5.30)	3.80(2.00–5.00)	-1.554	0.120
Cerebellum	7.65(4.50–10.80)	6.40(2.60–12.00)	-2.730	0.006^*^
Thalamus	8.70(4.40–22.70)	11.20(6.40–17.5)	-2.584	0.010^*^
White matter	2.60(2.00–5.70)	2.90(1.90–4.70)	-0.726	0.468
Gy matter	6.25(2.50–15.00)	7.95(5.00–11.90)	-2.582	0.010^*^

FDG, fluorodeoxyglucose; DEX, dexamethasone; CD, Cushing disease; SUV_mean_, mean standardized uptake value; ^18^F-FDG-PET/CT; ^18^ F-fluorodeoxyglucose positron emission tomography/computed tomography

### Qualitative analysis by diagnostic modalities for CD

**Fig. 4:**
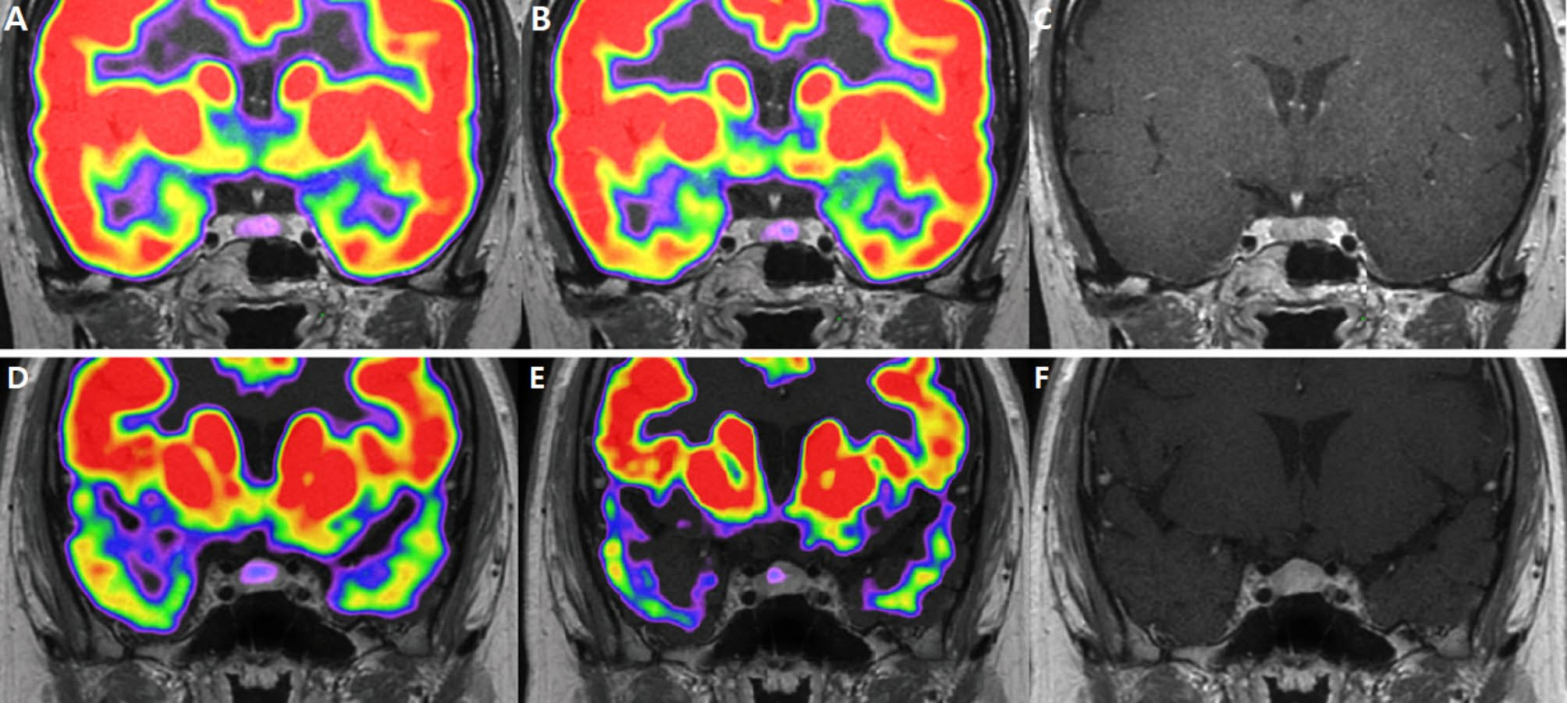
Images for corticotroph adenomas that appear different for localization in^18^F-FDG PET/CT. 9 mm sized adenoma in the left lateral wing of pituitary gland. It was found in the left lateral wing of the pituitary gland, showing an ^18^F-FDG uptake in the pituitary fossa with an SUV_mean_ of 1.04 and SUV_max_ of 1.07. However, after DEX suppression, the left side of the pituitary gland did not exhibit suppressed ^18^F-FDG uptake, with SUV_mean_ 1.05 SUV_max_ 1.14 (**A**). Co-registered baseline ^18^F-FDG PET/CT and MRI images. (**B**). Co-registered ^18^F-FDG PET/CT and MRI images after DEX suppression. (**C**). MRI image 2 mm pituitary adenoma was detected at the left lateral wing, showing diffuse FDG uptake in the pituitary fossa with an SUV_mean_ of 0.86 and SUV_max_ of 1.04. After DEX suppression, focal FDG uptake was observed, with SUV_mean_ 0.87 and SUV_max_ 0.98. (**D**). Co-registered baseline ^18^F-FDG PET/CT and MRI images. (**E**). Co-registered ^18^F-FDG PET/CT and MRI images after DEX suppression. (**F**). MRI image

The qualitative results of localizing pituitary adenomas in CD patients are shown in Table 4 and Fig. 4. Only 13 patients had BIPSS results. The success rates were 90.91% for MRI and 84.62% for BIPSS.

**Table 4:** Qualitative analysis by diagnostic modalities for CD

Sub No.	Sex, Age	Baseline ^18^F-FDG PET/CT		DEX suppression ^18^F-FDG PET/CT	MRI	Size(mm)	BIPSS
1	F,56		+	+	+	28.00	
2	F,36		+(right/midline)	+	+	18.00	
3	F,34		+	+	+	11.20	+
4	F,47		+	+	+	11.00	
5	F,41		+	+	+	11.00	
6	F,44		+	+	+	20.00	
7	F,42		+	+	+	9.70	
8	F,52		+(right/midline)	+	+	9.00	-
9	F,53		+	+	+	8.00	+
10	F,31		-	-	+	8.00	
11	F,37		+	+	+	7.70	+
12	F,46		+	+	+	6.50	+
13	F,55		+	+	+	6.00	+
14	F,33		-	-	+	5.50	-
15	F,38		+	+	+	5.00	
16	F,20		+(right/midline)	+	+	2.00	+
17	M,26		+	+	-	-	+
18	F,13		+	+	+	6.00	
19	F,14		-	-	+	5.50	+
20	F,51		+	+/-	+	4.00	+
21	F,17		-	-	+	2.00	+
22	M,19		-	-	-	-	+

CD, Cushing’s disease; Sub No., Subject number; ^18^F-FDG-PET/CT; ^18^ F-fluorodeoxyglucose positron emission tomography/computed tomography; DEX, dexamethasone; MRI, magnetic resonance imaging; BIPSS, bilateral inferior petrosal sinus sampling;

In baseline PET scans, the specialists agreed that pituitary adenomas were visible in 17 scans and not visible in 5 scans. They reached a consensus that the tumor was evident in two scans, but there was a discrepancy in their assessments of its location.

After DEX suppression, pituitary adenomas showed positive results in 16 scans and negative results in 5 scans. Specialists disagreed on the presence of pituitary adenomas in one case only.

Interobserver agreement for localizing adenomas was 0.872 (95%CI: 0.711, 1.033) for baseline PET/CT and 0.938 (95%CI: 0.762, 1.056) for post dexamethasone suppression PET/CT, confirming excellent interobserver agreements, and the result was judged reliable. Among the instances where both opinions agreed, there were no lesions that showed differences in visibility between scans before and after DEX administration. This meant that lesions were either consistently visible or invisible in both scenarios.

## Discussion

We found that DEX suppression did not improve localization of ACTH-secreting pituitary adenomas using ^18^F-FDG PET/CT. Further, it did not significantly affect FDG uptakes in adrenocorticotrophic adenomas or normal pituitaries in patients with CD. The decision to administer 8 mg dexamethasone was based on the standard high-dose DST, which is internationally recommended for differentiating between ectopic ACTH secretion and CD [26]. This test involved comparing serum cortisol levels at 8 am before and after a single dose of 8 mg dexamethasone administered at 11 pm. Suppression of the serum cortisol level to less than 50% of the baseline value indicated a diagnosis of CD [32, 33]. Previous studies have reported that the 8-mg DST has a sensitivity of 90%, specificity of 100%, accuracy of 96.8%, positive predictive value of 100%, and negative predictive value of 95.5% [34, 35]. Our use of 8 mg dexamethasone was based on the theory that orally administering dexamethasone at this dose can effectively suppress cortisol levels in ACTH-secreting pituitary tumors.

We expected that FDG uptake by corticotrophic adenomas would not decrease after DEX administration in patients with CD, and this change may improve the ability to discriminate the tumor location from surrounding tissues on ^18^F-FDG PET. The SUV_max_ of pituitary adenomas adjusted for the normal pituitary gland increased from 1.13 to 1.21. However, this change was not statistically significant, and the success rate of localizing corticotrophic adenomas using ^18^F-FDG PET was not significantly improved after DEX suppression. If the FDG uptake of adenomas changed lesser compared to that of surrounding normal tissues after DEX suppression, the tumor could be more easily visualized because of the difference.

In addition, we attempted to evaluate FDG uptakes in other brain areas (cerebellum, thalamus, white matter, and gray matter) according to DEX administration in CD patients. SUV_mean_ of the cerebellum decreased significantly, but that of the thalamus and gray matter increased after DEX suppression. DEX did not change FDG uptake in pituitary adenoma, normal pituitary, or white matter. In a previous study analyzing FDG PET in CD patients, researchers observed varying correlations between FDG uptake and blood cortisol concentration across different brain regions [35, 36]. Nevertheless, the examination did not include an analysis of FDG uptake in the pituitary gland. Additionally, no previous studies have explored the effects of high-dose dexamethasone suppression on brain glucose metabolism in individuals with CD. Further studies are needed to explain the change in FDG uptake after DEX administration in patients with CD.

^18^F-FDG PET/CT provides information regarding glucose metabolism in the brain in vivo and has been widely used to evaluate brain metabolism in clinical and research settings [37]. Here, the nadir cortisol level on the HD DST correlated with the SUV_mean_ and SUV_max_ of pituitary adenomas on PET scans after DEX suppression. Cortisol secretion activity is thought to be associated with metabolic activity, and DEX administration altered this. Cortisol levels and FDG uptake in other regions of the brain are correlated in patients with CD, but the correlation between cortisol and FDG uptake in the pituitary glands and/or corticotrophic adenomas themselves has not been discussed [35, 36]. In our study, cortisol levels did not show a correlation with FDG uptake of corticotrophic adenomas, but after DEX suppression FDG uptake showed a correlation with the nadir cortisol level on the HD DST. This indicated that tumors in which cortisol secretion was less suppressed by on the HD DST showed higher FDG uptake than tumors with lower cortisol levels on the HD DST.

Although many studies have analyzed FDG uptake of brain tumors, reference sites defined in each study varied without a uniform standard. Gray matter, white matter, or adjacent tumor tissue was defined as a reference site [38–40]. We measured SUV_mean_ of normal pituitary tissues, gray matter, white matter, thalamus, and cerebellum as possible references. We defined the SUV_mean_ of normal pituitary tissues as a reference because the localization of adenomas requires an apparent difference between the adenoma and surrounding tissues.

Use of fixed ROI to measure FDG uptake caused partial volume effect in this study. However, lesions smaller than 5 mm with intense FDG uptake may still show increased FDG uptake, especially in tumors, albeit with lower SUV values compared with the actual values [41]. This study was performed because pituitary adenomas smaller than 5 mm with higher FDG uptake than the surrounding pituitary parenchyma have been observed in routine clinical practice. To control for the partial volume effect, the analysis was performed again for tumors which were larger than 5 mm and had higher FDG uptake than the surrounding pituitary parenchyma, and the results remained unchanged.

PET/CT has been explored as an alternative to or combined with MRI for the localization of corticotrophic adenomas. ^18^F-FDG PET/CT has a limited role in CD diagnosis, but CRH stimulation can increase its success rate [22, 42]. This study is important for increasing the effectiveness of PET using DEX. In addition, data on DEX effect on brain metabolism in patients with CD will be important for future studies.

## Conclusions

DEX suppression did not improve the localization of ^18^F-FDG PET/CT in patients with CD. This is considered to have sufficient significance in an effort to increase the diagnostic value of ^18^F-FDG PET/CT.

### Electronic supplementary material

Below is the link to the electronic supplementary material.

Supplementary Material 1

Supplementary Material 2

## Data Availability

All datasets generated and/or analyzed during the current study are not publicly available but are available from the corresponding author upon reasonable request.

## List of Abbreviations

^18^F-FDG: ^18^F-fluorodeoxyglucose
PET/CT: Positron emission tomography/computed tomography
DEX: Dexamethasone
MRI: Magnetic resonance imaging
BIPSS: Bilateral inferior petrosal sinus sampling
CD: Cushing’s disease
SUV: Standardized uptake value
ACTH: Adrenocorticotropic hormone
CRH: Corticotrophin-releasing hormone
FDG: Fluorodeoxyglucose
24hr UFC: 24-hour urine free cortisol
ON DST: Overnight dexamethasone suppression test
HD DST: High-dose dexamethasone suppression test
SUV_mean_: Mean standardized uptake value
SUV_max_: Maximum standardized uptake value
P: Peripheral
IPS: Inferior petrosal sinuses
TSA: Transsphenoidal adenectomy
