# Supplementary material for: Dexamethasone suppression for 18F-FDG PET/CT to localize ACTH-secreting pituitary tumors
Source: Cancer Imaging. 2023 Sep 12;23:85. doi: 10.1186/s40644-023-00600-8 (PMC10496442; doi:10.1186/s40644-023-00600-8)
Supplement: Supplementary file 2 — Supplementary Material 2 [file 40644_2023_600_MOESM2_ESM.docx]

**Reviewer #1:**

1. There are issues with the presentation of the results of this negative study in this paper, as well as contextualisation of results within the field in the introduction.discussion. It should be considered what is of interest to the field from both a research and clinical perspective to argue for the utility of high dose dex-stimulated FDG PET/CT in the localisation of Cushing's Disease (CD).

FDG-PET/CT is already mentioned in CD guidelines (e.g Fleseriu et al Lancet Diabetes Endocrinol 2021; 9: 847-75) as an alternative localisation method in MRI negative tumours or alternatively FDG PET/MRI and so the novelty in the submitted paper is solely to demonstrate the role of dexamethasone suppression as having additional localisation value, as has been described for CRH-stimulation (Boyle et al Endocrine (2019) 65:155-165). The results of the paper from Kim et al are negative in this regard. The authors are overly optimistic in reporting the significance of their results.

Response

We appreciate the reviewer’s valuable comment. We agree that our study results were negative because dexamethasone suppression did not improve the success of localizing adrenocorticotrophic adenomas. Our primary focus was to determine whether dexamethasone suppression could enhance the diagnostic value of FDG PET in Cushing’s disease (CD). As a result, we have explicitly stated this negative outcome. Although this study did not improve the diagnostic utility of FDG PET, we aim to emphasize its significance as part of the clinical efforts to address the considerable need for preoperative tumor localization in patients with CD.

Changes: Abstract (Lines 45–55), Discussion (lines 331–335, 388–392), Conclusion (line 395–397)

2. The authors also omit to describe the local context in which patients received an FDG PET/CT before and after 8mg DST as this is not standard clinical practice internationally - was this a separate ethics protocol or representative of institutional practice?

Response

In this retrospective cohort study, we included all patients with Cushing's disease (CD) who underwent two sets of ^18^F-FDG-PET/CT before and after 8 mg dexamethasone suppression and sellar MRI before surgery. These patients were diagnosed based on international criteria. None of the patients was taking glucocorticoids. Moreover, the final diagnosis was confirmed using surgical pathology and clinical follow-up.

Administration of 8 mg dexamethasone was based on the standard high-dose dexamethasone suppression test (DST), which is internationally recommended to differentiate between ectopic ACTH secretion and CD.

High-dose DST involved comparing serum cortisol levels at 8 am before and after a single dose of 8 mg dexamethasone administered at 11 pm. If the serum cortisol level was suppressed to less than 50% of the baseline value, it indicated a diagnosis of CD. If suppression did not occur below that threshold, it indicated one of the other two causes of Cushing's syndrome: a glucocorticoid-secreting adrenal tumor or ectopic ACTH. Previous studies have reported that the 8 mg DST has a sensitivity of 90%, specificity of 100%, accuracy of 96.8%, positive predictive value of 100%, and negative predictive value of 95.5%.

Our use of 8 mg dexamethasone was based on the theory that orally administering dexamethasone at this dose can effectively suppress cortisol levels in ACTH-secreting pituitary tumors. We collected data as part of routine clinical care with approval from the ethics committee of our hospital. The requirement for written informed consent was waived due to the retrospective design of the study (institutional review board number: 2023-0110-001).

We have added this information to the Discussion (lines 336–342) section.

References

1. Fleseriu M, Auchus R, Bancos I, Ben-Shlomo A, Bertherat J, Biermasz NR, et al. Consensus on diagnosis and management of Cushing’s disease: a guideline update. Lancet Diabetes Endocrinol. 2021;9:847–75.

2. Dichek HL, Nieman LK, Oldfield EH, Pass HI, Malley JD, Cutler GB. A comparison of the standard high dose dexamethasone suppression test and the overnight 8-mg dexamethasone suppression test for the differential diagnosis of adrenocorticotropin-dependent Cushing’s syndrome. J Clin Endocrinol Metab. 1994;78:418–22.

3. Aytug S, Laws ER, Vance ML. Assessment of the utility of the high-dose dexamethasone suppression test in confirming the diagnosis of Cushing disease. Endocr Pract Off J Am Coll Endocrinol Am Assoc Clin Endocrinol. 2012;18:152–7.

4. Sriussadaporn S, Ploybutr S, Peerapatdit T, Plengvidhya N, Nitiyanant W, Vannasaeng S, et al. Nocturnal 8 mg dexamethasone suppression test: a practical and accurate test for identification of the cause of endogenous Cushing’s syndrome. Br J Clin Pract. 1996;50:9–13.

3. If the authors want to be able to make any claim that dex FDG PET/CT could have any additive clinical value on top of FDG PET/CT or MRI these images should be re-reviewed by blinded neuroradiologists/nuclear physicians and the data re-reported in this context. A retrospective review of non-standard clinical practice is not a strong enough study design.

Response:

We agree that the results of this study were negative, and we have changed the manuscript to present these negative results.

To enhance the precision of comparing the effectiveness of dexamethasone in localizing adenoma, two sets of PET images were anonymized and reevaluated. Two specialists independently interpreted the encoded PET image before and after 2 weeks of administering dexamethasone.

Interobserver agreement regarding image analysis was evaluated using κ statistics. κ values were categorized as follows: κ < 0.20 indicated poor agreement, κ of 0.21–0.40 indicated fair agreement, κ of 0.41–0.60 indicated moderate agreement, κ of 0.61–0.80 indicated good agreement, and κ > 0.81 indicated excellent agreement.

Interobserver agreement for localizing adenomas was 0.872 (95%CI: 0.711, 1.033) for baseline PET/CT and 0.938 (95%CI: 0.762, 1.056) for post dexamethasone suppression PET/CT, confirming excellent interobserver agreements, and the results were judged as reliable.

We have included this explanation in the Methods (lines 210–213) and Results (lines 317–319).

References

1. Jakobsson U, Westergren A. Statistical methods for assessing agreement for ordinal data. Scand J Caring Sci. 2005;19:427–31.

4. Indeed, for the single case where FDG PET/CT + dex suppression reportedly localised disease when the MRI was negative, the images shown to support this were subtle and it is not convincing that a blinded reviewer would have called the right-sided localisation. Additionally, in this case vignette BIPSS (which is standard to determine CD, with variable lateralisation accuracy) localised a right-sided tumour and the surgeon was able to visualise tumour tissue intraoperatively, so the additive value of dex-suppression FDG-PET/CT (especially the subtle findings shown) clinically is not clear in this case, which was meant to be an exemplar case.

Response

Currently, bilateral inferior petrosal sinus sampling (BIPSS) with CRH stimulation is the most reliable method since it offers the highest approachable sensitivity and specificity in confirming pituitary ACTH production exceeding 95%. However, the primary purpose of IPSS is to distinguish between CD and ectopic ACTH syndrome (EAS), because the presence of lateralization during this procedure is concordant with intraoperative biopsies in only 38.9% of Korean patients with CD. The current guideline does not recommend use of BIPSS to determine tumor lateralization.

BIPSS is an invasive procedure, its availability is limited in many areas and countries, and the rate of correct catheterization depends on the level of experience and expertise of the radiological team. Additionally, lateralization by BIPSS alone is indispensable for performing hemiphyectomy. Therefore, another noninvasive imaging modality is still needed for pituitary surgery, which selectively removes the tumor while preserving normal pituitary tissue, which is especially important for preserving hormonal balance.

References

1. Oldfield EH, Doppman JL, Nieman LK, Chrousos GP, Miller DL, Katz DA, et al. Petrosal sinus sampling with and without corticotropin-releasing hormone for the differential diagnosis of Cushing’s syndrome. N Engl J Med. 1991;325:897–905.

2. Zampetti B, Grossrubatscher E, Dalino Ciaramella P, Boccardi E, Loli P. Bilateral inferior petrosal sinus sampling. Endocr Connect. 2016;5:R12-25.

3. Lefournier V, Martinie M, Vasdev A, Bessou P, Passagia J-G, Labat-Moleur F, et al. Accuracy of bilateral inferior petrosal or cavernous sinuses sampling in predicting the lateralization of Cushing’s disease pituitary microadenoma: influence of catheter position and anatomy of venous drainage. J Clin Endocrinol Metab. 2003;88:196–203.

4. Moszczyńska E, Marczak E, Szalecki M, Kądziołka K, Roszkowski M, Zagata-Lesnicka P. The Effects of Sampling Lateralization on Bilateral Inferior Petrosal Sinus Sampling for Pediatric Cushing’s Disease-A Single Endocrinology Centre Experience and Review of the Literature. Front Endocrinol. 2021;12:650967.

5. Bonelli FS, Huston J, Carpenter PC, Erickson D, Young WF, Meyer FB. Adrenocorticotropic hormone-dependent Cushing’s syndrome: sensitivity and specificity of inferior petrosal sinus sampling. AJNR Am J Neuroradiol. 2000;21:690–6.

6. Lim JS, Lee SK, Kim SH, Lee EJ, Kim SH. Intraoperative multiple-staged resection and tumor tissue identification using frozen sections provide the best result for the accurate localization and complete resection of tumors in Cushing’s disease. Endocrine. 2011;40:452–61.

7. Frete C, Corcuff J-B, Kuhn E, Salenave S, Gaye D, Young J, et al. Non-invasive Diagnostic Strategy in ACTH-dependent Cushing’s Syndrome. J Clin Endocrinol Metab. 2020;105:3273–84

5. Negative studies do have publication value and given the rarity of Cushing's Disease and even rarer that these patients undergo FDG PET/CT it would be important to report the clinically relevant findings and describe FDG PET imaging biomarkers for CD. The numbers that are reported are low and comparison between the groups as well as determination of statistical significance is not corrected for multiple testing. It would be better to present descriptive results. / Given the small number of patients a more descriptive table with each patient listed with the imaging findings similar to Boyle et al Endocrine (2019) 65:155-165 e.g.

Response

We agree with your viewpoint that presenting the results in a descriptive manner would be better, particularly for studies involving a limited number of participants like our study, considering the inherent statistical limitations.

We have described the qualitative results of localizing pituitary adenomas in patients with CD in a new table.

Table 4. Qualitative analysis by diagnostic modalities for CD

| **Sub No.** | **Sex, Age** | **Baseline**  **^18^F-FDG PET/CT** | | **DEX suppression ^18^F-FDG PET/CT** | **MRI** | **Size(mm)** | **BIPSS** |
| --- | --- | --- | --- | --- | --- | --- | --- |
| 1 | F,56 | | + | + | + | 28.00 |  |
| 2 | F,36 | | + | + | + | 18.00 | – |
| 3 | F,34 | | + | + | + | 11.20 | + |
| 4 | F,47 | | + | + | + | 11.00 |  |
| 5 | F,41 | | + | + | + | 11.00 |  |
| 6 | F,44 | | + | + | + | 20.00 |  |
| 7 | F,42 | | + | + | + | 9.70 |  |
| 8 | F,52 | | +(right/midline) | + | + | 9.00 | – |
| 9 | F,53 | | + | + | + | 8.00 | + |
| 10 | F,31 | | – | – | + | 8.00 |  |
| 11 | F,37 | | + | + | + | 7.70 | + |
| 12 | F,46 | | + | + | + | 6.50 | + |
| 13 | F,55 | | + | + | + | 6.00 | + |
| 14 | F,33 | | – | – | + | 5.50 |  |
| 15 | F,38 | | + | + | + | 5.00 |  |
| 16 | F,20 | | +(right/midline) | + | + | 2.00 | + |
| 17 | M,26 | | + | + | – | – | + |
| 18 | F,13 | | + | + | + | 6.00 |  |
| 19 | F,14 | | – | – | + | 5.50 | + |
| 20 | F,51 | | + | +/– | + | 4.00 | + |
| 21 | F,17 | | – | – | + | 2.00 | + |
| 22 | M,19 | | – | – | – | – | + |

Only 13 patients had BIPSS results. The success rates were 90.91% for MRI and 84.62% for BIPSS.

In baseline PET scans, specialists agreed that pituitary adenomas were visible in 17 scans and not visible in 5 scans. They reached a consensus that the tumors were evident in two cases, but did not agree on its location. After DEX suppression, specialists concurred that pituitary adenomas showed positive results in 16 scans and negative results in 5 scans. Among the instances where both opinions agreed, there were no lesions that showed differences in visibility between scans before and after DEX administration. This meant that lesions were either consistently visible or invisible in both scenarios.

We have deleted the Results part of “Comparison of success rate of localizing pituitary tumors for CD” and table 3. Instead, we introduced a new table (table 4) and added a new subsection entitled “Qualitative analysis by diagnostic modalities for CD” and explained the findings in detail.

Changes: Results (Table 4, lines 308–322)

**6. Additional results that should be reported: Representative images and SUV max and SUVmean change of the 4 cases that were negative on baseline and positive on dex FDG-PET/CT should be displayed in a figure.**

Response

We presented two cases with disconcordanat localization in baseline PET scans after reevaluation of PET/CT. They reached a consensus that the tumors were visible in these two cases, but did not agree on its location.

First case had 9mm sized adenoma in the left lateral wing of pituitary gland on MRI. 18F-FDG uptake was shown in the pituitary fossa with an SUV_mean_ of 1.04 and SUV_max_ of 1.07. After DEX suppression, the left side of the pituitary gland did not exhibit suppressed 18F-FDG uptake, with SUV_mean_ 1.05 and SUV_max_ 1.14.

In second case, MRI revealed 2mm pituitary adenoma at the left lateral wing. Baseline PET/CT showed FDG uptake in the pituitary fossa with an SUV _mean_ of 0.86 and SUV_max_ of 1.04. After DEX suppression, focal FDG uptake was observed, with SUV_mean_ 0.87 and SUV_max_ 0.98.

We presented these two cases for figure 4 (below).

**New Figure 4. Images for adrenocorticotroph adenomas that appear different for localization in ^18^F-FDG PET/CT.**

Figure legend

9mm sized adenoma in the left lateral wing of pituitary gland. It was found in the left lateral wing of the pituitary gland, showing an 18F-FDG uptake in the pituitary fossa with an SUVmean of 1.04 and SUVmax of 1.07. However, after DEX suppression, the left side of the pituitary gland did not exhibit suppressed 18F-FDG uptake, with SUVmean 1.05 SUVmax 1.14.

A. Co-registered baseline 18F-FDG PET/CT and MRI images. B. Co-registered 18F-FDG PET/CT and MRI images after DEX suppression. C. MRI image.

2mm pituitary adenoma was detected at the left lateral wing, showing diffuse FDG uptake in the pituitary fossa with an SUVmean of 0.86 and SUVmax of 1.04. After DEX suppression, focal FDG uptake was observed, with SUVmean 0.87 and SUVmax 0.98. C. Co-registered baseline 18F-FDG PET/CT and MRI images. D. Co-registered 18F-FDG PET/CT and MRI images after DEX suppression. E. MRI image.

**7. Uncorrected SUVmax and SUVmean of the corticotroph adenomas and how this changed pre-post dex. This can be in addition to the corrected ratios already presented to show that the hypothesis that the ratio will increase with dex due to suppression of normal pituitary is not supported.**

**Response**

We have presented the uncorrected SUVs of corticotroph adenomas and normal pituitaries, as shown in the table below. Both adenomas and normal pituitaries were not significantly affected by DEX suppression.

| **SUV** | **Baseline**  **^18^F-FDG PET/CT** | **DEX suppression ^18^F-FDG PET/CT** | **Wilcoxon Z** | ***P* value** |
| --- | --- | --- | --- | --- |
| Adenoma SUV_max_ | 5.05 (3.20–8.60) | 4.70 (2.50–7.50) | –1.153 | 0.249 |
| Adenoma SUV_mean_ | 4.60 (2.80–8.30) | 4.50 (2.10–7.00) | –0.601 | 0.548 |
| Normal pitutiary SUV_max_ | 3.80 (2.40–5.70) | 4.30 (1.90–5.70) | –1.306 | 0.192 |
| Normal pituitary SUV_mean_ | 3.50 (2.40–5.30) | 3.80 (2.00–5.00) | –1.554 | 0.120 |

8. What was the Ki-67 of the tumours and was there any correlation with FDG uptake? Useful to report even if all were <1% as would be expected.

Response

In our analysis of clinical characteristics, we included the Ki-67 index. Table 1 displays the distribution of Ki-67 values among the patients, where 47.06% exhibited a Ki-67 index of 1–2, 35.29% had a Ki-67 index below 1, and 17.65% had a Ki-67 index ≥2.

Furthermore, we conducted additional analysis utilizing the Ki-67 index. However, we found no correlation between FDG uptake and Ki-67 index in both PET/CT scans, before and after dexamethasone administration (below table).

| **Spearman correlation coefficient (Rho), P-value** | |
| --- | --- |
| **SUVmax/mean** | |
| **Baseline** | **DEX suppression** |
| –0.107, P=0.693 | –0.189, P=0.484 |
| **SUVmean/SUVmean** | |
| **Baseline** | **DEX supression** |
| –0.161, P=0.553 | –0.119, P=0.660 |

Changes: Result (Table 1, lines 227–231)

10. Was the 1 patient who did not achieve remission post surgery the patient who did not have suppression on high dose DST? Did the surgery show a corticotroph adenoma or did this person have ectopic ACTH/CRH from a neuroendocrine neoplasm elsewhere and the adenoma was incidental? Did they have BIPSS? Should they be excluded from the analysis?

Response

A 36-year-old woman presented to our hospital with complaints of weight gain. During physical examination, she exhibited characteristic features of Cushing’s syndrome, such as central obesity with striae, a moon face, and a buffalo hump.

The test results revealed a 24-h urine free cortisol level of 316 µg/day, a basal cortisol level of 17.7 µg/dL, and an ACTH level of 90.34 pg/mL. Cortisol secretion was not suppressed on low-dose dexamethasone suppression test (LD DST) but was suppressed on high-dose dexamethasone suppression test (HD DST). A pituitary adenoma measuring 1.8 cm was detected in the left lateral wing of the pituitary gland with left cavernous sinus invasion in the sellar MRI. Additionally, abdomino-pelvic CT scan indicated diffuse thickening of both adrenal glands, suggesting bilateral adrenal hyperplasia. Bilateral Inferior Petrosal Sinus Sampling (BIPSS) confirmed a central tumor, but lateralization was inconclusive. 18-F FDG PET/CT before and after DEX suppression showed pituitary adenomas in the left side of the pituitary gland. Further, 111-In octreotide scan showed no other significant abnormal uptake suggestive of a neuroendocrine tumor, except for the pituitary adenoma. Surgical removal of the pituitary adenoma pathologically confirmed an ACTH-secreting pituitary tumor with 1–2% of Ki-67.

Based on the clinical judgment, the diagnosis was determined as ACTH-secreting pituitary tumor rather than ectopic Cushing's syndrome. Remission was not achieved due to cavernous sinus invasion. After gamma knife surgery, the patient is currently being treated with cabergoline and maintaining stable disease. We included this patient in the statistics because of the diagnosis of ACTH-secreting pituitary tumor.

11. The discussion should more accurately reflect that this was a negative study.

Response

We have significantly changed the manuscript to clearly present the negative results. We have also specified the negative results in the Abstract and Conclusion.

Original version of abstract

DEX suppression for PET may be helpful in locating microadenomas in patients with CD.

Revised version of abstract (line 54-55)

DEX suppression did not improve ^18^F-FDG PET/CT localization in patients with CD.

Original version of conclusion

DEX suppression may improve the success rate of microadenomas before ^18^F-FDG PET/CT in patients with CD. This is considered to have sufficient significance in an effort to increase the diagnostic value of ^18^F-FDG PET/CT.

Revised version of conclusion (line 395-397)

DEX suppression did not improve the localization of ^18^F-FDG PET/CT in patients with CD. This is considered significant in increasing the diagnostic value of ^18^F-FDG PET/CT.

**Reviewer #2:**

1. Thank you for your submission. While, I appreciate there may be interest in identifying the role of FDG PET in identifying and localising the pituitary gland adenomas, however I had difficulty to understand the rational for administration of one off 8 mg Dexamethasone orally and then 24 hours later repeating PET scan and expecting that it will make a significant difference in detecting and localising the adenoma from normal background tissue. As you correctly mentioned, there was positive correlation between size of tumour and the success rate of detection. This is because smaller lesions are below the resolution of PET scan.

Response

We agree that partial volume effect is a problem in this study. However, lesions smaller than 5 mm with intense FDG uptake may still show increased FDG uptake, especially in tumors, albeit with lower SUV values compared with the actual values. This study was performed because we have observed pituitary adenomas smaller than 5 mm with higher FDG uptake than the surrounding pituitary parenchyma in routine clinical practice.

Further, although lesions less than 5 mm are below the spatial resolution limit, the detection sensitivity of the PET scanner shows higher FDG uptake relative to the surrounding parenchyma. This study was proposed with the premise that FDG uptake in adenomas would not be suppressed by dexamethasone suppression; hence, higher FDG uptake should be seen in non-suppressed adenoma compared with suppressed normal pituitary adenoma.

To control for the partial volume effect, the analysis was performed again for tumors which were larger than 5 mm and had higher FDG uptake than the surrounding pituitary parenchyma. The results are presented in the table below. Concordant with our premise, dexamethasone suppression decreased FDG uptake in the normal pituitary gland, but not in the pituitary adenomas; this can be seen as a relative increase in FDG uptake in the pituitary adenoma/pituitary gland ratio. The occurrence of this trend could be because dexamethasone suppression differently influences glycolysis in adenomas and normal pituitary tissues.

| **SUV** | **Baseline**  **^18^F-FDG PET/CT** | **DEX suppression ^18^F-FDG PET/CT** | **Wilcoxon Z** | ***P* value** |
| --- | --- | --- | --- | --- |
| Adenoma SUV_max_ | 5.60(3.40–8.60) | 5.00(2.50–7.50) | -7.96 | 0.426 |
| Adenoma SUV_mean_ | 5.50 (3.30–8.30) | 4.95 (2.10–7.00) | –0.942 | 0.346 |
| Adenoma SUV_max_ /Normal pituitary SUV_mean_ | 1.36 (1.02–2.61) | 1.40 (1.03–2.65) | –1.022 | 0.307 |
| Adenoma SUV_mean_/Normal pituitary SUV_mean_ | 1.31 (1.04–2.52) | 1.33 (1.05–2.38) | –0.784 | 0.433 |

The tumors with size larger than 5 mm and SUV adjusted by normal pituitary＞1 are indicated by blue line. We have added the corresponding descriptions in the Results section.

Original Figure 2.

Revised Figure 3.

In addition, we have described the problem of partial volume effect in the Discussion section.

Changes: Results (Figure 3, lines 262–276), Discussion (lines 370–387).

2. Also it is not clear **how the region of interest of normal tissue and adenoma was chosen**. In addition, all these patients were suspected or proved to have adenoma. **There were no control group of people with normal gland to compare the difference in uptake.** Overall, it appears that PET has been more useful in those cases when MRI failed to detect the lesion but was avid on PET.

Response

ROI was drawn using MIM software. PET images were reviewed by an experienced radiologist. The pituitary gland was identified, and a circular ROI was drawn. We used a fixed ROI with a diameter of 3 mm for all lesions in all patients. The ROI was placed on the lesion with the highest FDG uptake. If there was no significantly increased FDG uptake, the same-sized circular ROI was drawn on the suspected adenoma location.

For the normal pituitary gland, the same-sized 3 mm ROI was used. We used the same-sized circular ROI on the normal pituitary tissues for reference.

Changes: Methods (lines 152–158)

3. Also it was not clear what variables have been controlled between baseline and after Dex PET as even slight variations in uptake time, etc could make some variations in SUV measurements etc. Controlling variables and doing exactly what was done in baseline PET for a such a small region in brain and in particular for sub-centimetre micro-adenomas requires matched case-control study to be able to conclude the difference.

Response

We appreciate the reviewer’s detailed comment. Although many studies have analyzed FDG uptake of brain tumors, the reference sites defined in each study vary without a uniform standard. Gray matter, white matter, or adjacent tumor tissue was defined as a reference site in these studies. We measured SUV_mean_ of normal pituitary tissues, gray matter, white matter, thalamus, and cerebellum as possible references. After dexamethasone suppression, normal pituitary gland and white matter showed no change in SUV_mean_, while cerebellum, thalamus, and gray matter showed significant changes in SUV_mean_.

According to a previous study that analyzed the cortisol level and FDG uptake in various brain regions in patients with Cushing’s disease, cortisol levels in the cerebellum and thalamus showed correlation with FDG uptake. However, there was no study on the change in FDG uptake after dexamethasone suppression in the brain of patients with Cushing’s disease exposed to long-term hypercortisolism. In this study, we defined the SUV_mean_ of normal pituitary tissues as a reference and summarized the SUV of the four areas we measured in table 3 (new table).

**(New) Table 3. Change in FDG uptake for reference sites after DEX suppression in the patients with CD**

| **SUV_mean_** | **Baseline**  **^18^F-FDG PET/CT** | **DEX suppression ^18^F-FDG PET/CT** | **Wilcoxon Z** | ***P* value** |
| --- | --- | --- | --- | --- |
| Normal pituitary gland | 3.50 (2.40–5.30) | 3.80 (2.00–5.00) | –1.554 | 0.120 |
| Cerebellum | 7.65 (4.50–10.80) | 6.40 (2.60–12.00) | –2.730 | 0.006^*^ |
| Thalamus | 8.70 (4.40–22.70) | 11.20 (6.40–17.5) | –2.584 | 0.010^*^ |
| White matter | 2.60 (2.00–5.70) | 2.90 (1.90–4.70) | –0.726 | 0.468 |
| Gray matter | 6.25 (2.50–15.00) | 7.95 (5.00–11.90) | –2.582 | 0.010^*^ |

Changes: Results (lines 293–300, Table 3) and Discussion (lines 351–361).

References

1. Chen W, Silverman DHS. Advances in evaluation of primary brain tumors. Semin Nucl Med. 2008;38:240–50.

2. Delbeke D, Meyerowitz C, Lapidus RL, Maciunas RJ, Jennings MT, Moots PL, et al. Optimal cutoff levels of F-18 fluorodeoxyglucose uptake in the differentiation of low-grade from high-grade brain tumors with PET. Radiology. 1995;195:47–52.

3. Meric K, Killeen RP, Abi-Ghanem AS, Soliman F, Novruzov F, Cakan E, et al. The use of 18F-FDG PET ratios in the differential diagnosis of common malignant brain tumors. Clin Imaging. 2015;39:970–4. Chen W. Clinical applications of PET in brain tumors. J Nucl Med Off Publ Soc Nucl Med. 2007;48:1468–81.

4. As you correctly identified, the neuroradiologist not being blinded to post-Dex PET already has caused selection bias and more detection rate of adenomas in those 4 cases after Dex PET.

Response

We appreciate the reviewer’s comment. To ensure precise comparison of the effectiveness of dexamethasone in localizing adenomas, the two sets of PET images were anonymized and reevaluated. Two specialists independently interpreted the encoded PET images before and 2 weeks after dexamethasone administration. These assessments were conducted independently.

The interobserver agreement for image analysis was assessed using κ statistics. κ values were categorized as follows: κ < 0.20 indicated poor agreement, κ of 0.21–0.40 indicated fair agreement, κ of 0.41–0.60 indicated moderate agreement, κ of 0.61–0.80 indicated good agreement, and κ > 0.81 indicated excellent agreement.

The results revealed an interobserver agreement of 0.872 (95%CI: 0.711, 1.033) for baseline PET/CT in localizing adenomas 0.938 (95%CI: 0.762, 1.056) for post-dexamethasone suppression PET/CT. These values indicate excellent interobserver agreements, confirming the reliability of the results.

Notably, there was no case where localization was successful in PET after dexamethasone administration in previous results but failed in the blinded reading. This explanation has been included in the Methods section.

Changes: Method (lines 210–213) and Results (lines 293–300).

References

1. Jakobsson U, Westergren A. Statistical methods for assessing agreement for ordinal data. Scand J Caring Sci. 2005;19:427–31.

5. The reduced SUV may not be explained by resistance and may have another explanation. The positive correlation between higher cortisol level and detection rate on PET is possibly explained by the size to tumour.

Response

We agree with your opinion that reduced SUV may not be explained by resistance. We have deleted our explanation about resistance and deleted the results part entitled “Homogeneity of ^18^F-FDG uptake of pituitary adenomas after DEX administration.” However, previous studies reported that adenoma size did not necessarily correlate with the degree of hypercortisolism in patients with CD.

We attempted to evaluate FDG uptakes in other brain areas (cerebellum, thalamus, white matter, and gray matter) according to DEX administration in patients with CD. SUV_mean_ of the cerebellum decreased significantly, but that of the thalamus and gray matter increased after DEX suppression. DEX did not change FDG uptake in pituitary adenoma, normal pituitary, or white matter. In a previous study analyzing FDG PET in patients with CD, researchers observed varying correlations between FDG uptake and blood cortisol concentration across different brain regions. Nevertheless, the examination did not include an analysis of FDG uptake in the pituitary gland. Additionally, no previous studies have explored the effects of high-dose dexamethasone suppression on brain glucose metabolism in individuals with CD. Furthers studies are needed to explain the change in FDG uptake after DEX administration in patients with CD.

Changes: Results (lines 293–300, Table 3) and Discussion (lines 374–379)

6. Explaining the way area of interest was chosen among normal tissue and adenoma in such a small region by highlighted images might be helpful.

Response

In response to comment #2 above, we have provided an explanation regarding the Region of Interest (ROI) setting in our study. Following the reviewer’s suggestion, we have included a visual illustration depicting the ROI settings and incorporated it into the text.

**Figure 1. Images of ROI for pituitary adenoma on ^18^F-FDG PET scan**

Example of ROI definition in pituitary adenomas of ^18^F-FDG PET scan of the patients with CD. We draw the fixed circular ROI with a 3-mm diameter for pituitary adenomas (red circle) and normal pituitary gland (green circle).

ROI, Reason of interest; ^18^F-FDG PET, ^18^F-fluorodeoxyglucose positron emission tomography; CD, Cushing disease.
